# Viral Polymerase Chain Reaction Testing of the Cerebrospinal Fluid: Comprehensive Care for Neonates With a Fever

**DOI:** 10.7759/cureus.72714

**Published:** 2024-10-30

**Authors:** Avrohom Levy, Ann Ross, Andrew Delle Donne

**Affiliations:** 1 Pediatrics, Jersey Shore University Medical Center, Neptune, USA; 2 Neonatology, Jersey Shore University Medical Center, Neptune, USA

**Keywords:** complete sepsis workup, febrile infant, febrile newborn, general pediatrics, lumbar puncture, neonatal fever, neonate, viral meningitis, viral meningitis neonate, viral meningitis sequelae

## Abstract

Neonates who develop fever have a high risk for serious infection, and while the standard of care involves performing a full sepsis evaluation, current guidelines do not include viral polymerase chain reaction (PCR) testing of the cerebrospinal fluid as a standard of care, which means that cases of viral meningitis can be missed. This case presentation discusses a neonate who had a fever at four days of life who then underwent a full sepsis evaluation. A respiratory viral panel demonstrated rhino/enterovirus positivity. A lumbar puncture was consistent with aseptic meningitis and newborn was prophylactically covered with acyclovir until the cerebrospinal fluid PCR confirmed enterovirus positivity and was negative for herpes simplex virus. The neonate experienced thrombocytosis and elevated transaminase levels, which resolved before discharge. Acyclovir was discontinued following a pediatric infectious disease consultation. The patient steadily improved, passed all screenings, and was discharged on day 16 with follow-up appointments arranged, including developmental pediatrics and repeat hearing screenings. Despite the viral panel indicating a respiratory virus, the presence of a respiratory virus in a neonate does not necessarily reduce the risk of a serious bacterial illness, especially in infants younger than 29 days. Viral meningitis can be associated with substantial complications and, so viral PCR testing of the cerebrospinal fluid allows clinicians to monitor for the development of severe disease and set up outpatient follow-ups for potential long-term sequelae of viral meningitis, such as neurodevelopmental delays.

## Introduction

Neonates who develop fever are at an increased risk for serious infection [1]. A fever is defined as a temperature ≥38.0°C [2]. Determining which neonates are at the highest risk for serious infection can be challenging [3]. For neonates who are ill-appearing without a clear cause, a full evaluation for sepsis is recommended, which includes blood and urine cultures, and a lumbar puncture to obtain a cerebrospinal fluid (CSF) bacterial culture, cell count, gram stain, glucose, protein, enterovirus polymerase chain reaction (PCR) testing, and herpes simplex virus (HSV) testing if there are risk factors present [2,4,5]. However, uncertainty arises when a neonate has a known viral etiology for their symptoms or when they are equivocal or well-appearing [1].

For neonates with a viral etiology, a positive viral test result may not sufficiently reduce the risk of a serious bacterial infection. First, it is important to recognize that the presence of a virus does not preclude the presence of bacteria. Moreover, viral infections have been shown to predispose neonates to bacterial superinfections [6]. However as an infant surpasses 28 days, the detection of respiratory viruses, the detection of respiratory viruses is associated with a significant reduction in the frequency of serious bacterial infection [7].

It is also important to consider viral meningitis. While bacteria are the most common causative agents of meningitis, viruses can also induce meningitis [8]. Enteroviruses are the most common causative agents of viral meningitis, but other viruses commonly known to cause meningitis include HSV, varicella-zoster virus, human herpesvirus 6, coxsackievirus-B, coronavirus disease SARS-CoV-2, cytomegalovirus, and Epstein-Barr virus [8,9].

While viral meningitis is generally self-limited, this is not always the case. Acute complications can include seizures, syndrome of inappropriate antidiuretic hormone, and increased intracranial pressure. Long-term complications can include seizure disorders, hydrocephalus, hearing loss, weakness/paralysis, cranial nerve palsies, learning disabilities, blindness, behavior disorders, and speech delays. Although these sequelae are generally more common in patients with bacterial meningitis, they can also occur in patients with viral meningitis [10]. There have been documented cases of complicated enterovirus meningitis with disseminated disease involving the liver, brain, and myocardium, which are associated with high mortality [11]. For survivors, long-term morbidity generally includes persistent hepatic and cardiac dysfunction and neurodevelopmental deficits [12]. 

This case presentation discusses a neonate who had a fever at four days of life in the setting of a known viral respiratory virus and underwent a full sepsis evaluation which demonstrated positivity for enterovirus via viral PCR testing of the cerebrospinal fluid. While current guidelines recommend performing a full sepsis evaluation, viral PCR testing of the cerebrospinal fluid is not a standard of care, which means that cases of viral meningitis can be missed. This case highlights the necessity of multidisciplinary care, including developmental pediatrics and hearing screenings, to ensure comprehensive management of the neonate's health beyond the immediate postnatal period.

This case was presented at the Hackensack Meridian Health Resident/Fellow Research Day on May 21st, 2024 with the title "To Tap or Not to Tap: Management of a Neonate with Fever and Known Viral Illness" [1].

## Case presentation

The patient is a female neonate born at 35+6 weeks gestation via elective repeat Cesarean section following preterm labor and spontaneous rupture of membranes to a G2P1 mother at a level II community hospital. The patient weighed 2530 grams at birth. The pregnancy was uncomplicated, and the mother received adequate prenatal care with all serologic screenings returning unremarkable results. Group B Streptococcus (GBS) status was negative.

At birth, the neonate experienced mild respiratory distress, likely secondary to transient tachypnea of the newborn, which was managed with continuous positive airway pressure for three days. Due to the combination of preterm labor, negative GBS status, and respiratory distress at birth, she was empirically started on ampicillin and gentamicin after collecting a blood culture. After 48 hours, with the culture remaining negative, antibiotics were discontinued. The blood culture ultimately resulted as no growth for five days (Table 1). The infant was successfully weaned to unassisted room air and tolerated all bottle feeds by mouth on day of life (DOL) 3.

**Table 1:** Blood and Surface Culture Results DOL: day of life

Laboratory Results	Result	Reference Range
Herpes Cultures (Rectal, Mouth, Eye)	Not Isolated	Not Isolated
Blood Culture DOL 1	No growth after 5 days	No growth
Blood Culture DOL 4	No growth after 5 days	No growth

On DOL 4, the neonate developed a fever of 101°F, followed by a second fever of 101°F several hours later. Although mildly tachycardic, she appeared otherwise well. A new evaluation for sepsis was initiated, including a repeat blood culture (Table 1), complete blood count (Table 2), metabolic panel (Table 2), and respiratory viral panel (RVP). An attempted lumbar puncture was unsuccessful. The RVP returned positive for rhino/enterovirus. Although stable on room air, the infant was transferred to a level III neonatal intensive care unit (NICU) for ongoing care due to the positive RVP results.

**Table 2:** Significant Hematology and Chemistry Laboratory Results DOL: day of life, ALT: alanine aminotransferase, AST: aspartate aminotransferase

Laboratory Results	Result DOL 1	Result DOL 4	Result at discharge	Reference Range
White Blood Cells	22.0	10.4	18.7	5.0 - 21.0 10*3/uL
Hemoglobin	14.0	17.1	17.5	13.5 - 21.5 g/dL
Platelet Count	311	381	1031	150 - 450 10*3/uL
Segmented Neutrophils	57 %	52%	24.6%	40.0 - 50.0 %
Neutrophils, Absolute	12.6	6.7	5.4	1.5 - 10.0 10*3/uL
Lymphocytes, Percent	25 %	25 %	65.4 %	36.0 - 46.0 %
Lymphocytes, Absolute	5.5	3.1	10.9	2.0 - 17.0 10*3/uL
C-Reactive Protein	0.36	1.86	0.6	<=0.30 mg/dL
AST	Not done	64	43	0 - 34 U/L
ALT	Not done	9	22	10 - 49 U/L

Upon arrival at the tertiary care NICU, the neonate remained febrile but appeared comfortable on unassisted room air, albeit less active than reported by the transferring hospital. A successful lumbar puncture yielded the following results: zero red blood cells, 390 white blood cells, protein of 116.7 mg/dL (reference range 15-45), and glucose of 44.1 mg/dL (reference range 60-80). The CSF was sent for culture, gram stain, and viral antigen PCR. The gram stain showed no organisms. Due to the elevated white blood cell count, acyclovir was initiated for concern for meningitis secondary to herpes simplex virus. Viral antigen PCR detected enterovirus in the CSF. The CSF study results are tabulated in Table 3.

**Table 3:** CSF Laboratory Results * The Meningitis Filmarray that was used can detect: Cytomegalovirus, Enterovirus, Herpes simplex virus 1 (HSV 1), Herpes simplex virus 2 (HSV 2), Human Herpesvirus 6 (HHV-6), and Human Parechovirus (HPeV), Varicella Zoster Virus (VZV) via viral antigen PCR testing.

Laboratory Results	Result	Reference Range
Appearance	Cloudy	Colorless
Xanthochromia	Yes	No
Red Blood Cells	0	0 /uL
White Blood Cells	390	0 - 30 /uL
Glucose	44.1	60.0 - 80.0 mg/dL
Protein	116.7	15.0 - 45.0
Segmented Neutrophils	23	0 - 8 %
Lymphocytes	17	5 - 35 %
Monocytes	60	50 - 90 %
Culture	No growth for 5 days	No growth
Herpes Simplex 1+2 PCR	Not Detected	Not Detected
Meningitis Filmarray*	Enterovirus Detected	Not Detected

Notable laboratory findings during this admission included thrombocytosis (which peaked at a platelet count of 1031) and transaminitis (aspartate aminotransferase and alanine aminotransferase peaking at 398 and 297, respectively on DOL 9), both which nearly normalized by discharge. These abnormalities were attributed to the viral infection. The results are tabulated in Table 2.

Following consultation with pediatric infectious disease, acyclovir was discontinued. The neonate did not develop any further fevers and was monitored in the NICU until she was able to nipple all feeds successfully and show consistent weight gain. She remained on unassisted room air and did not require any escalation of respiratory support. She passed all NICU screenings, including a hearing test. At discharge on DOL 16, follow-up appointments were arranged, including with developmental pediatrics and scheduled repeat hearing screens.

## Discussion

This case presents a neonate who developed a fever at four days of life in the context of a known viral respiratory infection. Despite the well-known protocol of performing a full sepsis workup for neonates with fevers, this case is significant as it emphasizes the necessity of a thorough sepsis workup even when a viral etiology is suspected or known. It demonstrates the importance of considering bacterial co-infections or other serious conditions, ensuring that no potential complications are overlooked. It also highlights the need for long-term follow-up, as monitoring for potential sequelae of viral meningitis is necessary for any neonate with fever. 

Previously, complete sepsis workups were performed on all infants that presented with fevers up to 90 days of age. However, changing bacteriology and advances in testing have allowed for more risk stratification [2]. Newer guidelines created discrete guidelines for neonates eight to 21 days of age, neonates 22 to 28 days of age, and infants older than 29 days. A fever in a neonate eight days old and younger warrants a full evaluation for sepsis without question [13,14]. These guidelines increasingly allow for more reliance on inflammatory markers as opposed to CSF studies, especially as a neonate gets older. For well-appearing neonates with a fever eight to 21 days old, it is still recommended that a complete sepsis workup be performed, while for neonates with a fever between 22 to 28 days old, clinicians can begin to rely more on inflammatory markers [2]. Laboratory values of inflammation are considered elevated at the following levels: (1) procalcitonin >0.5 ng/mL, (2) C-reactive protein (CRP) >20 mg/L, and (3) Absolute Neutrophil Count (ANC) at >4000 when used in conjunction with procalcitonin or >5200 when procalcitonin is unavailable. For these older infants, it is recommended to obtain blood and urine cultures regardless of inflammatory markers and to obtain a cerebrospinal fluid culture if inflammatory markers are elevated. If inflammatory markers are not elevated, obtaining a cerebrospinal fluid culture is up to the clinician’s discretion [2].

While viral meningitis can present as a benign self-limited infection, it can also present as severe disease [9]. Especially in neonates, viral meningitis can be associated with substantial neurological complications and significant mortality [15]. Acute and chronic complications of viral meningitis are similar to those of bacterial meningitis and can include seizures, syndrome of inappropriate antidiuretic hormone, and increased intracranial pressure; as well as more long-term issues including hydrocephalus, and neurodevelopmental deficits [1,10-12].

Excluding HSV, the treatment for severe disease is largely supportive care. When it comes to HSV, acyclovir is indicated for both mild and severe disease. Regarding other viruses, viral meningitis is generally self-limiting, and supportive care is sufficient [16]. However, when it comes to severe disease, intravenous immune globulin has been shown to have potential and can improve survival [11,12]. Similarly, pleconaril, an oral viral capsid inhibitor with activity against picornaviruses, including most enteroviruses and many rhinoviruses, has been shown to have the potential to improve survival as well [17].

Evaluating CSF can be beneficial in differentiating between viral and bacterial meningitis [1]. Viral meningitis typically demonstrates a lower white blood cell count, with a normal glucose concentration, and a normal to slightly elevated protein concentration. Neutrophil predominance is common in the early stages of infection and switches to a lymphocytic predominance as the illness progresses [11]. In this case, the patient exhibited a high white blood cell count, along with a slightly decreased glucose concentration and an elevated protein level in the cerebrospinal fluid. Initially, a neutrophilic predominance was observed, which subsequently transitioned to a lymphocytic predominance. Although this presentation does not align perfectly with classical viral meningitis, the laboratory findings are nonetheless consistent with a diagnosis of viral etiology. Follow-up for both viral and bacterial meningitis should be assessed for hearing loss, psychosocial problems, neurologic sequelae, and developmental delays [18]. In this case, follow-up appointments for developmental pediatrics and scheduled repeat hearing screens were arranged at discharge, to ensure comprehensive management of the neonate's health beyond the immediate postnatal period.

## Conclusions

In conclusion, this case highlights the critical need for the implementation of viral PCR testing in cerebrospinal fluid as a standard practice in the evaluation of febrile neonates. Current guidelines primarily focus on bacterial infections, but guidelines that include viral PCR testing can lead to more efficient management. The ability to rapidly and accurately identify viral pathogens not only aids in the timely diagnosis of viral meningitis but also helps to differentiate it from bacterial infections, thereby reducing unnecessary antimicrobial therapy and its associated risks. Given the potential complications of viral meningitis, including neurodevelopmental delays, the use of viral PCR testing facilitates closer monitoring and more effective outpatient follow-up for affected neonates. By integrating viral PCR testing into clinical protocols, we can enhance patient outcomes and ensure a comprehensive approach to the management of neonates.
